# Association of Posttraumatic Stress Disorder With Mental Stress–Induced Myocardial Ischemia in Adults After Myocardial Infarction

**DOI:** 10.1001/jamanetworkopen.2020.2734

**Published:** 2020-04-14

**Authors:** Bruno B. Lima, Muhammad Hammadah, Brad D. Pearce, Amit Shah, Kasra Moazzami, Jeong Hwan Kim, Samaah Sullivan, Oleksiy Levantsevych, Tené T. Lewis, Lei Weng, Lisa Elon, Lian Li, Paolo Raggi, J. Douglas Bremner, Arshed Quyyumi, Viola Vaccarino

**Affiliations:** 1 Department of Epidemiology, Rollins School of Public Health, Emory University, Atlanta, Georgia; 2 Division of Cardiology, Department of Medicine, Emory University School of Medicine, Atlanta, Georgia; 3 Atlanta VA Medical Center, Decatur, Georgia; 4 Department of Biostatistics and Bioinformatics, Rollins School of Public Health, Emory University, Atlanta, Georgia; 5 Mazankowski Alberta Heart Institute, University of Alberta, Edmonton, Alberta, Canada; 6 Departments of Psychiatry, Emory University School of Medicine, Atlanta, Georgia; 7 Behavioral Sciences and Radiology, Emory University School of Medicine, Atlanta, Georgia

## Abstract

**Importance:**

Posttraumatic stress disorder (PTSD) is prevalent among patients who survived an acute coronary syndrome and is associated with adverse outcomes, but the mechanisms underlying these associations are unclear.

**Objective:**

To evaluate the association of PTSD with mental stress–induced myocardial ischemia among individuals who survived a myocardial infarction (MI).

**Design, Setting, and Participants:**

This cross-sectional study included 303 patients aged 18 to 60 years enrolled from a university-affiliated network. Participants had a verified history of MI within 8 months. Data were collected from June 2011 to March 2016, and data analysis was conducted from March to June 2019.

**Exposures:**

A clinical diagnosis of PTSD (lifetime and current) was obtained using the Structured Clinical Interview from the *Diagnostic and Statistical Manual of Mental Disorders* (Fourth Edition), and PTSD symptom subscales were assessed with the civilian version of the PTSD Symptom Checklist.

**Main Outcomes and Measures:**

Patients received technetium 99m–labeled sestamibi myocardial perfusion imaging at rest, with mental stress (ie, a speech task) and conventional stress (ie, exercise or pharmacologic). A summed difference score (ie, the difference between stress and rest scores) was used to assess ischemia under both stress conditions.

**Results:**

Among 303 participants (148 [48.8%] women; 198 [65.3%] African American; mean [SD] age, 51 [7] years), the prevalence of PTSD was 14.5% (44 patients). Patients with PTSD had a higher rate of ischemia with mental stress than those without PTSD (12 of 44 [27.3%] vs 38 of 259 [14.7%]; *P* = .04) and more than twice the mean number of ischemic segments (1.2 [95% CI, 0.5-1.8] vs 0.5 [95% CI, 0.3-0.7]; *P* < .001), but there was no difference in conventional stress ischemia (10 of 44 [22.7%] vs 60 of 259 [23.2%]; *P* = .91). Increasing levels of PTSD symptoms were associated with higher odds of ischemia with mental stress (adjusted odds ratio [OR] per 5-point score increase, 1.18; 95% CI 1.04-1.35; *P* = .01) but not with conventional stress (adjusted OR per 5-point score increase, 1.05; 95% CI, 0.92-1.21; *P* = .47). Reexperiencing trauma was the symptom cluster most robustly associated with the presence of ischemia with mental stress (adjusted OR per 5-point score increase, 1.87; 95% CI 1.21-2.91; *P* = .005), followed by avoidance and numbing (adjusted OR per 5-point score increase, 1.51; 95% CI, 1.07-2.14; *P* = .02).

**Conclusions and Relevance:**

In this study of young and middle-aged individuals with MI, with a large representation of women and patients from racial/ethnic minority groups, PTSD was associated with the development of myocardial ischemia with mental stress. A higher ischemic response to mental stress represents a potential pathway associating PTSD with adverse outcomes after MI.

## Key Points

**Question:**

Is posttraumatic stress disorder (PTSD) associated with mental stress–induced myocardial ischemia among patients with stable coronary artery disease?

**Findings:**

In this cross-sectional study of 303 young and middle-aged individuals who have survived a recent myocardial infarction, patients with PTSD were more likely to develop myocardial ischemia with mental stress compared with those who did not have PTSD.

**Meaning:**

Myocardial ischemia provoked by psychological stress could be a pathway for increased cardiovascular risk among patients with PTSD.

## Teaser

This cohort study evaluates the association of posttraumatic stress disorder (PTSD) with mental stress–induced myocardial ischemia among individuals who survived a myocardial infarction.

## Introduction

Posttraumatic stress disorder (PTSD) is prevalent in individuals who have survived a myocardial infarction (MI)^1,2,3^ and is associated with recurrent events and mortality.^4,5,6,7^ The precise mechanisms through which PTSD may increase the risk of adverse cardiovascular outcomes are not fully understood. Poor health behaviors,^8^ inflammation,^9,10^ and abnormal autonomic function^11,12^ have been linked to both PTSD and coronary heart disease (CHD) but whether these factors fully explain the increased risk associated with PTSD is debatable.^3^

Another possible mechanism mediating the association between PTSD and adverse outcomes after MI is that patients with MI and comorbid PTSD might be susceptible to developing myocardial ischemia during psychologic stress. People with PTSD have a dysregulated hypothalamic-pituitary-adrenal axis and sympathetic adrenal-medullary system,^13,14,15^ potentially making them more vulnerable to stress-induced adverse cardiovascular consequences. Repeated hypothalamic-pituitary-adrenal axis and sympathetic adrenal-medullary system activation by stressful exposures, such as trauma reminders in PTSD, could lead to long-term microvascular dysfunction, epicardial disease, endothelial injury, and inflammation,^16,17,18,19,20^ increasing the propensity for mental stress–induced myocardial ischemia (MSIMI).^21,22^ Mental stress–induced myocardial ischemia is a common phenomenon in patients with stable CHD^21,23^ and is associated with twice the risk of cardiac events and death.^24^ This phenomenon can be studied experimentally in the laboratory by using a standardized mental stress test, but, to our knowledge, it has never been evaluated with respect to PTSD.

Symptoms of PTSD are divided in 3 distinct subscales, as follows: (1) reexperiencing the traumatic events, such as through dreams, flashbacks, and intrusive, distressing thoughts; (2) avoidance of trauma reminders and numbing of emotions; and (3) hyperarousal, such as disturbed sleep, difficulty concentrating, irritability, and hypervigilance.^25^ These symptom subscales may index different vulnerability pathways for adverse health consequences among patients with PTSD. For instance, in a previous study,^10^ we demonstrated that the reexperiencing trauma symptom cluster was associated with the exacerbated inflammatory response to mental stress seen in patients with comorbid PTSD and MI.

In a sample of young and middle-aged men and women with a recent MI, we sought to determine whether the presence of PTSD and the severity of PTSD symptoms were associated with MSIMI. We also evaluated whether hemodynamic responses (eg, blood pressure and heart rate) and vascular responses to mental stress differed by PTSD status, given that these could help explain the association of PTSD with MSIMI. Finally, we explored whether specific PTSD symptom clusters were associated with MSIMI, with the hypothesis that the reexperiencing trauma symptom cluster would be most strongly associated with MSIMI. As a control condition, we evaluated the same patients to see whether PTSD was associated with conventional stress–induced myocardial ischemia (CSIMI), provoked by either pharmacologic or exercise stress testing.

## Methods

### Study Design and Participants

Between June 2011 and March 2016, we enrolled 313 patients with recent MI (159 [50.8%] men; 154 [49.2%] women) in the Myocardial Infarction and Mental Stress Study 2. The methods of this study have been previously described.^23^ Patients with MI were recruited from the pool of patients who were admitted with a documented MI in the previous 8 months (ie, index MI) at Emory University–affiliated hospitals in Atlanta, Georgia, and who were aged 18 to 60 years at the time of screening. The diagnosis of MI (type 1) was verified by medical record review based on standard criteria of a troponin level increase and electrocardiogram changes.^26^ Patients were selected so that approximately 50% of the sample would be women. Angiographic data were obtained from the coronary angiogram reports associated with the index MI, and the severity of coronary artery disease was quantified using the Gensini score.^27^ Scoring took into account revascularized vessels for the patients that received such interventions by examining angiograms after the intervention. This research was approved by the Emory University institutional review board. Written informed consent was obtained from all participants enrolled in the study. This study followed the reporting requirements of the Strengthening the Reporting of Observational Studies in Epidemiology (STROBE) reporting guideline.^28^ For a complete description of the study population, see eAppendix 1 in the Supplement.

### Measurements

#### Assessment of PTSD

Current (ie, past month) history of PTSD was assessed using the Structured Clinical Interview for *Diagnostic and Statistical Manual of Mental Disorders* (Fourth Edition) (*DSM-IV*), which provides a clinical diagnosis of psychiatric disorders.^29^ Symptoms of PTSD were assessed using the civilian version of the PTSD Symptom Checklist, a 17-item scale that assesses PTSD symptoms in the past month.^30^ The 17 items from the PTSD Symptom Checklist scale were further used to calculate the severity of symptoms during the last month on the following 3 subscales: reexperiencing trauma (items 1-5); avoidance and numbing (items 6-12); and arousal (items 13-17).^29^ We also assessed depressive symptoms using the Beck Depression Inventory, a 21-item self-administered scale,^31^ and general perceived stress with the Perceived Stress Scale.^32^

#### Mental Stress Testing Procedure

Patients were tested in the morning after a 12-hour fast. In a quiet, dimly lit, temperature-controlled (ie, 21-23 °C) room, vital signs were measured after a 30-minute rest period, and mental stress was induced by a standardized public speaking task.^33^ For a complete description of the mental stress procedure, see eAppendix 2 in the Supplement.

#### Conventional Stress Testing

On a separate day within 1 week of the mental stress test, conventional stress testing was performed using the Bruce protocol or, when contraindicated, pharmacologic testing with regadenoson. The radioisotope injection was given at peak exertion during the exercise test or immediately after the regadenoson injection.

#### Vascular Testing

Before and after the mental stress test, peripheral vasoconstriction using Endo-PAT 2000 (Itamar Medical) and endothelium-dependent brachial artery flow-mediated vasodilation (FMD) were measured, as described previously.^21,34,35,36^ For a complete description of the vascular function measurements, see eAppendix 3 in the Supplement.

#### Single-Photon Emission Computed Tomography Imaging

Myocardial perfusion imaging with technetium 99m–labeled sestamibi–single-photon emission computed tomography (SPECT) was performed at rest, after mental stress, and after conventional stress according to standard protocols.^37^ For a complete description of the SPECT imaging interpretation, see eAppendix 4 in the Supplement.

#### Other Measures

High-sensitivity C-reactive protein levels were measured from serum samples collected prestress (n = 554) using the electrochemiluminescence system by MesoScale and the SECTOR Imager 2400 (Meso Scale Diagnostics). The lower limit of detection was 1.33 × 10^7^ mg/dL (to convert to milligrams per liter, multiply by 10), and interassay and intraassay coefficients of variations were 3.06% and 2.33%, respectively.

### Statistical Analysis

Patient characteristics were compared by PTSD status, and differences were tested using *t* tests or Mann-Whitney-Wilcoxon tests for continuous variables and χ^2^ tests for categorical variables. The natural logarithmic transformation was used for nonnormally distributed variables (ie, peripheral arterial tonometry ratio and Gensini score). Logistic regression analysis was used to derive odds ratios (ORs) for the association of PTSD symptom severity with the presence of MSIMI or CSIMI after adjusting for CHD risk factors. Continuous variables were inspected for deviations from linearity in the logistic model. All analyses were conducted before and after adjusting for possible confounding factors considered a priori*,* including demographic factors (ie, sex, age, race, years of education), lifestyle and clinical risk factors (ie, smoking, body mass index, diabetes, hypertension, ST-segment myocardial infarction, dyslipidemia, ejection fraction, left ventricular ejection fraction, high-sensitivity C-reactive protein level, Gensini coronary artery disease severity score, resting perfusion defect severity [ie, the SPECT summed rest score], statin use, clopidogrel use, *DSM*-*IV* diagnosis for depression, and antidepressant use). Statistical significance was set at *P* < .05, and all tests were 2-tailed. All statistical analyses were conducted using SAS statistical software version 9.4 (SAS Institute).

## Results

Of 303 patients (148 [48.8%] women; 198 [65.3%] African American; mean [SD] age, 51 [7] years) with stable CHD included in the analysis, 44 (14.5%) had history of PTSD as determined by a Structured Clinical Interview for *DSM-IV* interview, and the median (interquartile range [IQR]) PTSD symptom score was 50 (34-59) in this group vs 25 (20-34) in the group without PTSD (*P* < .001) (Table 1). Patients with PTSD, compared with those without PTSD, were more likely to be African American (36 [81.8%] vs 162 [62.5%]; *P* = .04), to have a history of depression (33 [75.0%] vs 74 [28.6%]; *P* < .001), to have a higher median (IQR) Perceived Stress Scale score (23 [17-26] vs 15 [9-21]; *P* < .001), and to have a higher median (IQR) Beck Depression Inventory score (20 [12-25] vs 8 [4-16]; *P* < .001). In terms of medications, patients with PTSD, compared to those without, were less likely to take clopidogrel (25 [56.8%] vs 185 [71.4%]; *P* = .04) and statins (31 [70.5%] vs 224 [86.5%]; *P* = .01). A comparison of resting hemodynamics and vascular function between patients with and without PTSD showed that patients with PTSD and MI had a lower mean (SD) resting FMD (3.7% [2.0%] vs 3.9% [2.8%]; *P* = .01), but other resting hemodynamic and vascular parameters were similar. Otherwise, the groups shared similar clinical characteristics.

**Table 1.** Characteristics of the Study Population by PTSD Status

Characteristic	No. (%)		*P* value
	With PTSD (n = 44)	No PTSD (n = 259)	
Demographic characteristics			
Age, mean (SD), y	50 (8)	51 (6)	.29
Women	27 (61.4)	121 (46.7)	.09
African American	36 (81.8)	162 (62.5)	.04
Married or living with a partner	16 (36.4)	115 (44.4)	.32
Education, mean (SD), y	13 (2)	14 (3)	.06
PTSD Symptom Checklist score, median (IQR)			
Total	50 (34-59)	25 (20-34)	<.001
Reexperiencing trauma symptoms	13 (9-18)	6 (5-10)	<.001
Hyperarousal symptoms	18 (10-19)	10 (6-11)	<.001
Avoidance and numbing symptoms	14 (12-24)	8 (8-14)	<.001
Psychosocial risk factors			
History of major depression	33 (75.0)	74 (28.6)	<.001
Beck Depression Inventory score, median (IQR)	20 (12-25)	8 (4-16)	<.001
Perceived Stress Scale score, median (IQR)	23 (17-26)	15 (9-21)	<.001
Cardiovascular risk factors			
BMI, mean (SD)	32 (9)	31 (7)	.36
Smoking status			
Never	19 (43.2)	118 (45.6)	.61
Past	12 (27.3)	82 (31.7)	
Current	13 (29.5)	59 (22.8)	
Baseline HsCRP, mean (SD), mg/dL	0.8 (1.1)	0.6 (0.9)	.14
Diabetes	16 (36.4)	81 (31.3)	.50
Hypertension	40 (90.9)	207 (79.9)	.08
Dyslipidemia	33 (75.0)	211 (81.5)	.32
Clinical characteristics			
STEMI	14 (31.8)	74 (28.6)	.93
Summed rest score, mean (SD)	3.7 (6.9)	3.7 (6.1)	.62
Ejection fraction, mean (SD), %	51 (12)	51 (12)	.93
Gensini angiographic severity score, median (IQR)	0 (0-12)	2.5 (0-11)	.91
History of MI before index MI	14 (31.8)	48 (18.5)	.03
History of heart failure	7 (15.9)	23 (8.9)	.15
History of CABG	10 (22.7)	52 (20.1)	.69
History of PCI	26 (59.1)	185 (71.4)	.10
Medications			
Statins	31 (70.5)	224 (86.4)	.01
β-blockers	39 (88.6)	219 (84.6)	.55
Aspirin	34 (77.3)	210 (81.1)	.49
Clopidogrel	25 (56.8)	185 (71.4)	.04
ACE inhibitors	26 (59.1)	117 (45.2)	.10
Antidepressants	8 (18.2)	43 (16.6)	.81
Resting hemodynamics			
Systolic blood pressure, mean (SD), mm Hg	138 (25)	134 (21)	.30
Diastolic blood pressure, mean (SD), mm Hg	78 (14)	75 (12)	.15
Heart rate, mean (SD), bpm	68 (13)	66 (11)	.37
RPP, mean (SD), beat × mm Hg/min/1000	9 (3)	9 (2)	.34
Resting vascular function			
RHI, mean (SD)	1.7 (0.5)	1.8 (0.6)	.29
FMD, mean (SD), %	3.7 (2.0)	3.9 (2.8)	.01

Abbreviations: ACE, angiotensin-converting enzyme; BMI, body mass index (calculated as weight in kilograms divided by height in meters squared); bpm, beats per minute; CABG, coronary artery bypass graft; FMD, flow-mediated vasodilation; HsCRP, high-sensitivity C-reactive protein; IQR, interquartile range; MI, myocardial infarction; PCI, percutaneous coronary intervention; PTSD, posttraumatic stress disorder; RHI, reactive hyperemia index; RPP, rate pressure product; STEMI, ST-segment elevation MI.

SI conversion factor: To convert HsCRP to milligrams per liter, multiply by 10.

### Hemodynamic, Vascular, and Subjective Response to Mental and Conventional Stress

Overall, hemodynamic responses (ie, blood pressure and heart rate) to mental stress were similar in patients with PTSD compared with patients without PTSD (Table 2). However, patients with PTSD showed a larger mean (SD) FMD decrease with mental stress compared with patients without PTSD (−2.0 [1.6] percentage points vs *−*1.7 [2.2] percentage points; *P* = .02), indicating a more pronounced transient endothelial dysfunction with mental stress. In contrast, there were no significant differences between groups in peripheral arterial tonometry ratio, subjective distress, or reactive hyperemia index changes with mental stress. During conventional stress, patients with and without PTSD demonstrated similar exercise tolerance. Overall, there were no differences in any of the hemodynamic and vascular responses to conventional stress between patients from these groups.

**Table 2.** Unadjusted Differences in Hemodynamic, Vascular, and Subjective Responses to Stress by PTSD Status (N = 303)

Response	With PTSD	No PTSD	*P* value
**Mental stress**			
Change in hemodynamic responses, mean (SD)^a^			
Blood pressure, mm Hg			
Systolic	43 (17)	45 (15)	.70
Diastolic	29 (12)	28 (11)	.61
Heart rate, bpm	25 (17)	25 (14)	.92
RPP, beat × mm Hg/min/1000	6 (4)	6 (3)	.45
Vascular responses, mean (SD)			
RHI			
Poststress	1.6 (5.7)	1.7 (6.1)	.72
Change^b^	−1.0 (4.5)	−0.3 (5.2)	.25
FMD, %			
Poststress	1.7 (1.9)	2.2 (2.4)	.07
Change^b^	−2.0 (1.6)	−1.7 (2.2)	.02
PAT ratio^c^	0.8 (0.4)	0.8 (0.5)	.41
Change in Subjective Units of Distress scale^d^	31 (33)	24 (30)	.14
**Conventional stress**			
Change in hemodynamic responses, mean (SD)^a^			
Blood pressure, mm Hg			
Systolic	64 (25)	58 (22)	.15
Diastolic	11 (12.5)	12 (14)	.33
Heart rate, bpm	72 (28)	72 (27)	.97
RPP, beat × mm Hg/min/1000	15 (7)	16 (6)	.69
Maximum work level, median (IQR), MET	8.1 (6.7-10.4)	9.4 (7.0-10.8)	.29
Minutes on treadmill, median (IQR), min	7.1 (5.1-8.4)	7.7 (5.9-9.5)	.17
Vascular responses, mean (SD)			
RHI			
Poststress	1.7 (5.0)	1.7 (5.3)	.61
Change^b^	−0.02 (5.3)	−0.35 (5.7)	.69
FMD, %			
Poststress	2.6 (2.2)	2.7 (2.2)	.65
Change^b^	−1.2 (2.2)	−1.1 (2.7)	.13

Abbreviations: bpm, beats per minute; FMD, flow-mediated vasodilation; IQR, interquartile range; MET, metabolic equivalents of task; PAT, peripheral arterial tonometry; PTSD, posttraumatic stress disorder; RHI, reactive hyperemia index; RPP, rate pressure product.

^a^ Difference between maximum value during stress and minimum value during rest.

^b^ Difference between posttest and pretest values; a negative value indicates worsened vascular function.

^c^ Ratio of pulse wave amplitude during the speaking task over the resting baseline, with lower values indicating greater vasoconstriction.

^d^ Difference between posttest and pretest values; a positive value indicates higher distress with mental stress.

### Myocardial Perfusion

Conventional stress testing was performed with a treadmill test among 214 patients (70.6%), and the remaining 89 had a pharmacologic stress test. Incidences of MSIMI and CSIMI were 16.5% (50 patients) and 23.1% (70 patients), respectively (Table 3). Among patients who developed MSIMI, 20 (40.0%) also developed CSIMI; MSIMI was present in 38 patients (14.7%) without PTSD and 12 patients (27.3%) with PTSD (*P* = .04) (Table 3). In contrast, there was no difference in CSIMI between patients with and without PTSD (10 patients [22.7%] vs 60 [23.2%]; *P* = .91). Results remained consistent when examining the mean (SD) number of ischemic segments (1.2 [95% CI, 0.5-1.8] vs 0.5 [95% CI, 0.3-0.7]; *P* < .001) and when expressing ischemia as a percentage of ischemic myocardium.

**Table 3.** Unadjusted Differences in Myocardial Ischemia Measures by PTSD Status

Measure	No. (%)		*P* value
	With PTSD (n = 44)	No PTSD (n = 259)	
**Mental stress**			
Myocardial ischemia	12 (27.2)	38 (14.7)	.04
Ischemic segments, mean (95% CI), No.	1.2 (0.5-1.8)	0.5 (0.3-0.7)	<.001
LV with inducible ischemia, %			
0	32 (72.7)	200 (77.2)	<.001^a^
>0 to <5	3 (6.8)	29 (11.2)	
≥5 to <10	3 (6.7)	16 (6.2)	
≥10	6 (13.6)	10 (3.9)	
**Conventional stress**			
Myocardial ischemia	10 (22.7)	60 (23.2)	.91
Ischemic segments, mean (95% CI), %	1.1 (0.5-1.7)	1.1 (0.8-1.3)	.95
LV with inducible ischemia, %			
0	33 (75.0)	167 (64.5)	.26^a^
>0 to <5	1 (2.3)	28 (10.8)	
≥5 to <10	2 (4.5)	32 (12.4)	
≥10	8 (18.2)	28 (10.8)	

Abbreviations: PTSD, posttraumatic stress disorder; LV, left ventricle.

^a^ Mantel-Haenszel test for linear trend.

Consistent with these findings, increasing levels of PTSD symptoms were associated with higher occurrence of MSIMI but not CSIMI (Table 4). For each 5-point increase in PTSD symptoms, the unadjusted OR for MSIMI was 1.11 (95% CI, 1.01-1.23; *P* = .04). These associations were unchanged after controlling for demographic factors, lifestyle factors, and clinical risk factors (adjusted OR, 1.18; 95% CI, 1.04-1.35; *P* = .01) but not with conventional stress (adjusted OR, 1.05; 95% CI, 0.92-1.21; *P* = .47).

**Table 4.** Association of Posttraumatic Stress Disorder Severity With Myocardial Ischemia With Mental Stress or Conventional Stress

Model	Myocardial ischemia with mental stress		Myocardial ischemia with conventional stress	
	5-Point score increase, OR (95% CI)	*P* value	5-Point score increase, OR (95% CI)	*P* value
Unadjusted	1.11 (1.01-1.23)	.04	1.03 (0.94-1.13)	.51
Adjusted for sociodemographic and lifestyle factors^a^	1.12 (1.00-1.24)	.05	1.00 (0.90-1.11)	.99
Adjusted for sociodemographic and lifestyle factors as well as CHD risk factors, medical history, and CHD severity^b^	1.13 (1.01-1.28)	.04	1.00 (0.89-1.12)	.98
Adjusted for sociodemographic, lifestyle, and health factors plus psychosocial factors^c^	1.18 (1.04-1.35)	.01	1.05 (0.92-1.21)	.47

Abbreviations: CHD, coronary heart disease; OR, odds ratio.

^a^ Adjusted for age, race (black vs nonblack), years of education, current smoking, and body mass index.

^b^ Adjusted for hypertension, dyslipidemia, diabetes mellitus, ST-segment myocardial infarction, left ventricular ejection fraction, high-sensitivity C-reactive protein level, Gensini coronary artery disease severity score, resting perfusion defect severity (summed rest score), and statin and clopidogrel use.

^c^ Adjusted for *Diagnostic and Statistical Manual of Mental Disorders* (Fourth Edition) diagnosis for depression and antidepressant use.

Next, we analyzed PTSD symptoms according to 3 *DSM-IV* symptom subscales, ie, reexperiencing trauma; avoidance and numbing; and arousal (Table 5). Reexperiencing trauma was the symptom cluster most robustly associated with MSIMI (adjusted OR per 5-point increase in symptom score, 1.87; 95% CI, 1.21-2.91; *P* = .005), followed by avoidance and numbing (adjusted OR per 5-point increase in symptom score, 1.51; 95% CI, 1.07-2.14; *P* = .02). Arousal symptoms were not significantly associated with increased odds of MSIMI (adjusted OR per 5-point increment in symptom score, 1.42; 95% CI, 0.93-2.16; *P* = .10).

**Table 5.** Association of Subscales of Posttraumatic Stress Disorder Symptoms With Myocardial Ischemia With Mental Stress or Conventional Stress

Subscale^a^	Myocardial ischemia with mental stress		Myocardial ischemia with conventional stress	
	5-Point score increase, OR (95% CI)	*P* value	5-Point score increase, OR (95% CI)	*P* value
Reexperiencing trauma				
Unadjusted	1.53 (1.12-2.08)	.007	1.12 (0.84-1.51)	.43
Adjusted^b^	1.87 (1.21-2.91)	.005	1.06 (0.77-1.44)	.73
Avoidance and numbing				
Unadjusted	1.27 (1.00-1.62)	.05	1.03 (0.82-1.29)	.80
Adjusted^b^	1.51 (1.07-2.14)	.02	1.00 (0.78-1.26)	.94
Arousal				
Unadjusted	1.20 (0.88-1.63)	.24	1.14 (0.87-1.50)	.34
Adjusted^b^	1.42 (0.93-2.16)	.10	1.06 (0.79-1.42)	.69

Abbreviation: OR, odds ratio.

^a^ Subscales of posttraumatic stress disorder symptoms were calculated from the PTSD Symptom Checklist scale.

^b^ Adjusted for age, race (black vs nonblack), years of education, current smoking, body mass index, hypertension, dyslipidemia, diabetes mellitus, ST-segment myocardial infarction, left ventricular ejection fraction, high-sensitivity C-reactive protein level, Gensini coronary artery disease severity score, and resting perfusion defect severity (summed rest score), *Diagnostic and Statistical Manual of Mental Disorders* (Fourth Edition) diagnosis for depression, and antidepressant, statin, and clopidogrel use.

To assess potential vascular mechanisms of differences in MSIMI for patients with vs without PTSD, we examined whether resting hemodynamic and vascular measures and their changes with mental stress explained differences in MSIMI. However, no vascular or hemodynamic measurements were associated with ischemia with either mental or conventional stress by PTSD status (data not shown).

## Discussion

In a well-characterized sample of survivors of a recent MI, we showed that patients with PTSD were nearly twice as likely as those without PTSD to develop MSIMI, but not CSIMI. Furthermore, worse PTSD symptoms, especially on the symptom subscales of reexperiencing trauma and avoidance and numbing, were associated with an increase in the odds of developing MSIMI, but not CSIMI. These findings were independent of clinical and behavioral risk factors. While we did not find differences in hemodynamic responses and peripheral vasoconstriction during mental stress by PTSD status, patients with MI and PTSD had a lower FMD at rest and a more pronounced FMD decline after mental stress, indicating impairment in endothelial function.

Posttraumatic stress disorder is a disabling mental health disorder that is prevalent among patients who have survived an acute coronary syndrome or other acute life-threatening illness.^1,38^ Although it remains uncertain whether PTSD was a consequence of the acute coronary syndrome or was preexisting, the prevalence of PTSD in our study (ie, 14.5%) was similar to what was previously reported (ie, 16%).^1,2,3^

In previous studies of patients with MI or other acute coronary syndromes, PTSD was independently associated with a doubling of recurrent events and mortality, but the underlying mechanisms remained speculative.^3^ In this study, we provided insights into a risk mechanism that associates PTSD with MSIMI. Notably, PTSD was specifically associated with ischemia provoked by mental stress, not ischemia provoked by exercise or pharmacologic stress. These results suggest a sensitivity for patients with MI and PTSD toward ischemia in response to an emotional stressor rather than a physical stressor. Turner et al^7^ studied a predominantly male veteran population and reported an independent association of PTSD with myocardial ischemia induced by exercise stress testing. This divergence from our results could be owing to differences in study populations, given that all participants in our study had a recent MI while the participants in the study by Turner et al^7^ were a mixed sample of outpatients. The higher risk status of participants in this study could have attenuated differences in the presence of CSIMI between those with and without PTSD.

It is unlikely that reverse causation is an explanation for the association between PTSD and MSIMI, ie, that a higher severity index MI could cause both PTSD symptoms and a propensity for ischemia. There were no differences in clinical severity indicators between patients with and without PTSD, except for a higher prevalence of an MI prior to the index event and a tendency for lower use of cardiovascular medications among those with PTSD. Furthermore, the association between PTSD and MSIMI persisted even after adjusting for indicators of coronary artery disease severity, such as Gensini score, left ventricular ejection fraction, resting perfusion defects, high-sensitivity C-reactive protein level, and statin and clopidogrel use.

The symptom cluster most strongly associated with MSIMI was reexperiencing trauma, followed by avoidance and numbing, while hyperarousal was not significantly associated with MSIMI. This is consistent with our prior results,^10^ which associated symptoms of reexperiencing trauma with higher interleukin-6 response to mental stress in the same patient population. However, whether inflammation is directly associated with MSIMI is still unclear, given that previous literature has yielded mixed results.^22,39^

In addition to upregulating the transcription of proinflammatory immune response genes,^22,40^ activation of the sympathetic nervous system and withdrawal of the parasympathetic nervous system with mental stress can cause myocardial ischemia through an increase in heart rate, blood pressure, and peripheral vasoconstriction.^21^ However, in our study, there were no differences in hemodynamic responses or most vascular responses to mental stress between patients with and without PTSD, suggesting that these mechanisms of MSIMI may not play a large role in our results.

Poor endothelial function is a potential mechanism for the higher occurrence of MSIMI in patients with MI and PTSD. We found that patients with MI and PTSD had lower baseline FMD than those without PTSD, which is consistent with previous results in the general population.^21,41,42^ Furthermore, we showed that patients with PTSD had a larger decline in FMD with mental stress compared with patients without PTSD. Increased sympathetic tone during mental stress induces vasoconstriction of peripheral arteries, especially in the microvascular bed, and a decline in the nitric oxide–mediated vasodilatory response through α1-adrenergic receptor activation; an endothelin 1–dependent pathway may also be implicated.^21,41,42^ This differs from physical exertion, which causes a vasodilator response through a β2-adrenoceptor mechanism. Thus, patients with MI and PTSD may experience a more substantial reduction in nitric oxide in the vascular wall in response to emotional stress than those without PTSD. Endothelial dysfunction measured peripherally with FMD is not a strong risk factor for MSIMI,^43^ and in our study, peripheral vascular measures and their changes with mental stress did not explain differences in MSIMI by PTSD status. However, these findings do not exclude a mechanistic role of endothelial dysfunction at the coronary level, given that the coronary microvascular response to mental stress is primarily endothelium dependent.^44^ Although SPECT myocardial perfusion imaging primarily assesses epicardial, not microvascular, coronary disease, mental stress can also cause coronary epicardial vasoconstriction.^44^

While the underlying mechanism linking PTSD to MSIMI in patients who have survived MI still needs clarification, the implications of our results are that during daily life, patients with MI and PTSD may undergo repeated episodes of mental stress and neurohormonal activation through reexperiencing symptoms, which may result in episodes of silent myocardial ischemia. The latter, in conjunction with stress-induced transient endothelial dysfunction, epicardial coronary artery vasoconstriction,^44^ and increased inflammation, can contribute to increased risk of adverse outcomes.

### Limitations and Strengths

This study has limitations. The extent of inducible ischemia with mental stress was overall relatively mild, and because we lacked outcome data, the clinical significance of our findings needs further study. It is possible that the speech task we used for mental stress testing (although an established method in studies of patients with CHD where ischemia is assessed with technetium 99m–labeled sestamibi perfusion imaging) may have limited sensitivity or specificity for the assessment of MSIMI and other cardiovascular responses to acute stress in individuals with PTSD. Potentially, a mental stress test based on trauma recall could elicit greater responses in individuals with PTSD. However, if this were an issue, it would likely bias the estimates toward the null. Furthermore, our study was conducted at a single institution, and generalizability to other populations and settings need further investigation.

Despite these limitations, this remains, to our knowledge, the only study of MSIMI and PTSD among patients with a recent history of MI. The experimental design, the inclusion of a control condition of conventional stress testing, and the large number of women and individuals from racial/ethnic minority groups are unique strengths of our investigation. Also, we used myocardial perfusion imaging, which remains the criterion standard for assessment of ischemia and has important advantages for mental stress testing because the radioisotope is injected during the speech task and provides a snapshot of perfusion at the time of acute stress.

## Conclusions

In this study of a well-characterized sample of survivors of a recent MI, PTSD was associated with a 2-fold higher likelihood of developing ischemia with mental stress but not with conventional stress. The PTSD symptom cluster of reexperiencing trauma showed the strongest association with MSIMI. Our results highlight MSIMI as a novel potential pathway of risk in PTSD and are consistent with the hypothesis that traumatic reminders could lead to repeated episodes of MSIMI in daily life, leading to worse cardiovascular outcomes in patients with MI and PTSD.

## Notes

Supplement. **eAppendix 1. **Study Design and Participants **eAppendix 2. **Mental Stress Procedure **eAppendix 3. **Vascular Function Measurements **eAppendix 4. **Myocardial Perfusion Imaging and SPECT Imaging Interpretation **eReferences.**
