# Supplementary material for: Association of Posttraumatic Stress Disorder With Mental Stress–Induced Myocardial Ischemia in Adults After Myocardial Infarction
Source: JAMA Netw Open. 2020 Apr 14;3(4):e202734. doi: 10.1001/jamanetworkopen.2020.2734 (PMC7156990; doi:10.1001/jamanetworkopen.2020.2734)
Supplement: Supplement. — eAppendix 1. Study Design and Participants eAppendix 2. Mental Stress Procedure eAppendix 3. Vascular Function Measurements eAppendix 4. Myocardial Perfusion Imaging and SPECT Imaging Interpretation eReferences. [file jamanetwopen-3-e202734-s001.pdf]

Supplementary Online Content

Lima BB, Hammadah M, Pearce BD, et al. Association of posttraumatic stress disorder with mental stress–induced myocardial ischemia in adults after myocardial infarction. *JAMA Netw Open*. 2020;3(4):e202734. doi:10.1001/jamanetworkopen.2020.2734

eAppendix 1. Study Design and Participants
eAppendix 2. Mental Stress Procedure
eAppendix 3. Vascular Function Measurements
eAppendix 4. Myocardial Perfusion Imaging and SPECT Imaging Interpretation
eReferences.

This supplementary material has been provided by the authors to give readers additional information about their work.

© 2020 Lima BB et al. *JAMA Network Open.*

eAppendix 1. Study Design and Participants

Subjects were excluded if they had a severe comorbid medical or psychiatric disorder that could interfere with the study results, such as cancer, renal failure, severe uncontrolled hypertension, current alcohol or substance abuse, bipolar disorder or schizophrenia; if they were pregnant or breastfeeding; or if they were currently using immunosuppressant or psychotropic medications other than antidepressants. MI patients were also excluded if they had unstable angina, acute MI or decompensated heart failure within the previous week; if they weighed over 450 pounds (due to weight bearing limits of the nuclear stress test equipment); and if it was deemed to be unsafe by study cardiologists to withhold anti-ischemic medications for 24 hours before the testing.

Of 313 CHD patients in the MIMS2 dataset, 10 patients had missing PTSD status data. Thus, a total of 303 participants were included in the current study. This research was approved by the Emory University Institutional Review Board. Written informed consent was obtained from all patients enrolled in the study.

Demographic information was obtained using standardized questionnaires. Previous medical history (diabetes, hypertension, previous MI) and medication use (e.g. aspirin, beta blockers) were obtained by study nurses or physicians through medical history, clinical examinations and by reviewing medical records. Height and weight were measured during the clinical exam and used to calculate body mass index (BMI, kg/m^2).

eAppendix 2. Mental Stress Procedure

Briefly, patients were asked to imagine a situation in which a close relative had been mistreated in a nursing home. Patients were given 2 min to prepare and 3 min to deliver a speech in front of an evaluative audience. Blood pressure and heart rate were recorded throughout. Mental stress testing was performed by trained and experienced staff to ensure standardization of the test.[1]

Hemodynamic parameters, including systolic blood pressure (SBP), diastolic blood pressure (DBP), and heart rate (HR), were recorded using the IntelliSense Professional Digital Blood Pressure Monitor (OMRON, Japan). The rate-pressure product was calculated as SBP x HR. Hemodynamic parameters were recorded every 5 min during the resting period, every 1 min during the mental stress, and every 5 min during the recovery period. Hemodynamic responses to mental stress were calculated as the difference between the maximum value of each hemodynamic parameter during the speech minus the minimum resting value during the rest period.

© 2020 Lima BB et al. *JAMA Network Open.*

eAppendix 3. Vascular Function Measurements

We assessed peripheral vasoconstriction and microvascular function using the Endo-PAT2000 (Itamar Medical), which measures finger pulse volume amplitude (PVA), reflecting peripheral blood volume changes using volume plethysmography technology as described previously.[2-4] PVA signals detected by a finger probe were filtered, amplified, and analyzed in an operator-independent manner. The baseline PVA was determined by averaging the last 3 minutes of recording that preceded the mental stress test. The stress amplitude was determined as the lowest PVA during the speaking period. The peripheral arterial tonometry (PAT) ratio was then calculated by the software as the ratio of PVA during the speaking task over the resting baseline, with a ratio <1 signifying a vasoconstrictive response. Before and 30 minutes after the mental stress test, we also used the EndoPAT device to assess the digital reactive hyperemia index (RHI), a measurement of peripheral microvascular function. For this test, PVA is obtained in resting condition and during reactive hyperemia, which is elicited by the release of an upper arm blood pressure cuff inflated to suprasystolic pressure for 5 minutes. The RHI is then calculated as the ratio of post-deflation to baseline pulse amplitude in the hyperemic finger divided by the ratio in the contralateral finger. This metric is calculated by a computer algorithm and has been associated with cardiovascular outcomes. Before and 30 minutes after the mental stress test, patients also underwent measurement of flow-mediated vasodilation (FMD) of the brachial artery via bimode ultrasound to assess endothelial function using standard methodology.[2,5] In our laboratory, the mean difference in FMD between assessments performed in 11 subjects on consecutive days was 1.26% (SD 0.76), with a correlation coefficient of 0.75. The mean difference in FMD between 2 readings of the same 11 measurements was 0.82% (SD 0.48, $r = 0.97$). Reproducibility of cardiovascular

© 2020 Lima BB et al. *JAMA Network Open.*

responses with mental stress in our laboratory have been published.[3] A sample (n=22) of participants underwent repeated measures (including systolic blood pressure, diastolic blood pressure, heart rate, rate-pressure product, and PAT ratio) at two time points (within eight weeks) using the same study protocol. Bland-Altman plots showed excellent reproducibility of all measures.

© 2020 Lima BB et al. *JAMA Network Open.*

eAppendix 4. Myocardial Perfusion Imaging and SPECT Imaging Interpretation

Subjects with MI underwent 3 single-photon emission computed tomography myocardial perfusion imaging scans after injection of sestamibi radiolabeled with Technetium-99m ([^{99m}Tc]), at rest, during mental stress, and during conventional stress at the dose of 10 to 14 mCi of ^{99m}Tc for rest imaging and 30 to 40 mCi for stress imaging based on weight. Testing was done in 2 separate days up to 1 week apart on a dedicated single-photon emission computed tomography camera (Philips Cardio MD) with attenuation correction. We withheld antiischemic medications for 24 hours before testing following standard nuclear cardiology protocols. On the mental stress day, [^{99m}Tc]sestamibi was injected 1 minute after onset of the public speech task. On the conventional stress day, subjects underwent a standard Bruce protocol or (if unable to exercise) a pharmacological stress test with regadenoson (Abbott). The radioisotope injection was given at peak exertion or immediately after the regadenoson injection.

Studies were interpreted by 2 experienced readers without prior knowledge of severity of CHD or other patient medical history. Discrepancies in interpretation of SPECT images were resolved by consensus. Rest and stress images were visually compared for number and severity of perfusion defects using a 17-segment model. Each segment was scored from 0 to 4, with 0 being normal uptake and 4 no uptake. Ischemia was defined as a new impairment with a score ≥2 in any segment, or as worsening of a pre-existing impairment by at least 2 points if in a single segment, or by at least 1 point if in 2 or more contiguous segments.[6] In addition to individual segment scores, we calculated summed scores in a conventional fashion, including a summed stress score, a summed rest score and a summed difference score, the latter representing a semi-quantitative measure of inducible ischemia. For exercise and pharmacological stress, presence of ischemia was defined as a summed difference score ≥4.[7] For MSIMI, a summed

© 2020 Lima BB et al. *JAMA Network Open.*

difference score ≥3 is typically used as evidence of ischemia.[8] Mental stress-induced ischemia was defined as a summed difference score ≥3, and conventional stress ischemia as a summed difference score ≥4, as previously described.[2,4,9,10] We also calculated the percentage of myocardium with resting perfusion defects as: (summed rest score ÷ 68) × 100, and percent of ischemic myocardium as: (summed difference score ÷ 68) × 100.[10]

Cardiovascular responses with mental stress using our protocol have shown excellent reproducibility as previously published.[3] Reproducibility of mental stress induced myocardial ischemia with Tc99m sestamibi SPECT using our protocol has also been demonstrated.[3,11]

© 2020 Lima BB et al. *JAMA Network Open.*

eReferences

1. Hammadah M, Al Mheid I, Wilmot K, et al. The Mental Stress Ischemia Prognosis Study: Objectives, Study Design, and Prevalence of Inducible Ischemia. *Psychosom Med.* 2017;79(3):311-317.
2. Hammadah M, Alkhoder A, Al Mheid I, et al. Hemodynamic, catecholamine, vasomotor and vascular responses: Determinants of myocardial ischemia during mental stress. *Int J Cardiol.* 2017;243:47-53.
3. Sullivan S, Hammadah M, Al Mheid I, et al. Sex Differences in Hemodynamic and Microvascular Mechanisms of Myocardial Ischemia Induced by Mental Stress. *Arterioscler Thromb Vasc Biol.* 2018;38(2):473-480.
4. Vaccarino V, Sullivan S, Hammadah M, et al. Mental Stress-Induced-Myocardial Ischemia in Young Patients With Recent Myocardial Infarction: Sex Differences and Mechanisms. *Circulation.* 2018;137(8):794-805.
5. Lima BB, Hammadah M, Kim JH, et al. Association of Transient Endothelial Dysfunction Induced by Mental Stress With Major Adverse Cardiovascular Events in Men and Women With Coronary Artery Disease. *JAMA Cardiol.* 2019.
6. Holly TA, Abbott BG, Al-Mallah M, et al. Single photon-emission computed tomography. *J Nucl Cardiol.* 2010;17(5):941-973.
7. Hachamovitch R, Berman DS, Shaw LJ, et al. Incremental prognostic value of myocardial perfusion single photon emission computed tomography for the prediction of cardiac death: differential stratification for risk of cardiac death and myocardial infarction. *Circulation.* 1998;97(6):535-543.
8. Ramachandruni S, Fillingim RB, McGorray SP, et al. Mental stress provokes ischemia in coronary artery disease subjects without exercise- or adenosine-induced ischemia. *J Am Coll Cardiol.* 2006;47(5):987-991.
9. Hammadah M, Sullivan S, Pearce B, et al. Inflammatory response to mental stress and mental stress induced myocardial ischemia. *Brain Behav Immun.* 2018;68:90-97.
10. Vaccarino V, Wilmot K, Al Mheid I, et al. Sex Differences in Mental Stress-Induced Myocardial Ischemia in Patients With Coronary Heart Disease. *J Am Heart Assoc.* 2016;5(9).
11. Kim CK, Bartholomew BA, Mastin ST, Taasan VC, Carson KM, Sheps DS. Detection and reproducibility of mental stress-induced myocardial ischemia with Tc-99m sestamibi SPECT in normal and coronary artery disease populations. *J Nucl Cardiol.* 2003;10(1):56-62.

© 2020 Lima BB et al. *JAMA Network Open.*
